# Synergy of Information in Multimodal Internet of Things Systems—Discovering the Impact of Daily Behaviour Routines on Physical Activity Level

**DOI:** 10.3390/s25185619

**Published:** 2025-09-09

**Authors:** Mohsen Shirali, Zahra Ahmadi, Jose Luis Bayo-Monton, Zoe Valero-Ramon, Carlos Fernandez-Llatas

**Affiliations:** 1 Department of Computing Science and Engineering (INGI), Université Catholique de Louvain (UCLouvain), 1348 Louvain-la-Neuve, Belgium; 2 Research Centre for Information Systems Engineering (LIRIS), KU Leuven, 1000 Brussels, Belgium; zahra.ahmadi@kuleuven.be; 3 Process Mining 4 Health Lab–SABIEN-ITACA Institute, Universitat Politècnica de València, 46022 Valencia, Spain; jobamon@itaca.upv.es (J.L.B.-M.); cfllatas@itaca.upv.es (C.F.-L.); 4 Department of Clinical Sciences, Intervention and Technology (CLINTEC), Karolinska Institutet, 17177 Stockholm, Sweden

**Keywords:** behaviour modelling, physical activity monitoring, process mining, synergy, Internet of Things, multimodality

## Abstract

**Background and Objective:** The intricate connection between daily behaviours and health necessitates robust monitoring, particularly with the advent of Internet of Things (IoT) systems. This study introduces an innovative approach that exploits the synergy of information from various IoT sources to assess the alignment of behavioural routines with health guidelines. The goal is to improve the readability of behaviour models and provide actionable insights for healthcare professionals. **Method:** We integrate data from ambient sensors, smartphones, and wearable devices to acquire daily behavioural routines by employing process mining (PM) techniques to generate interpretable behaviour models. These routines are grouped according to compliance with health guidelines, and a clustering method is used to identify similarities in behaviours and key characteristics within each cluster. **Results:** Applied to an elderly care case study, our approach categorised days into three physical activity levels (Insufficient, Sufficient, Desirable) based on daily step thresholds. The integration of multi-source data revealed behavioural variations not detectable through single-source monitoring. We demonstrated that the proposed visualisations in calendar and timeline views aid health experts in understanding patient behaviours, enabling longitudinal monitoring and clearer interpretation of behavioural trends and precise interventions. Notably, the approach facilitates early detection of behaviour changes during contextual events (e.g., COVID-19 lockdown and Ramadan), which are available in our dataset. **Conclusions:** By enhancing interpretability and linking behaviour to health guidelines, this work signifies a promising path for behavioural analysis and discovering variations to empower smart healthcare, offering insights into patient health, personalised interventions, and healthier routines through continuous monitoring with IoT-driven data analysis.

## 1. Introduction

The study of human behaviour has gained increasing attention, particularly within health and well-being in recent years. These fields acknowledge the close connection between human behaviour patterns and health conditions in a way that an improper lifestyle or changes in daily habits can be early indicators of diseases [1,2]. For instance, physical inactivity stands out as a significant risk factor for certain diseases such as obesity, diabetes, and cardiovascular problems, underscoring the importance of accurately measuring activity levels to identify individuals at risk [3]. Many research studies also have proved the utility of monitoring daily activities to detect abnormal behaviours and discover deviations from typical routines, signalling early stages of health problems (e.g., dementia, Alzheimer’s disease, osteoporosis, arthritis) [4,5,6] or timely detection of hazardous incidents occurrence (such as falls) [7].

Consequently, in light of this relationship between human activities and behaviours with health conditions, global health associations and organisations regularly release guidelines to encourage people to achieve healthier physical conditions through lifestyle strengthening. These guidelines offer specific recommendations for individuals, mainly concerning quantities and frequencies of particular activities. In this context, by understanding people’s daily activities and behaviour routines, healthcare professionals can ensure individuals engage in the right activities that are conducive to their health at the right times. Therefore, a comprehensive insight into a patient’s health condition is a prerequisite for healthcare experts if they aim to provide effective healthcare services.

The professionals can keep track of individuals’ activities to inspect whether their behaviour aligns with the health guidelines. This information also allows the implementation of more precise interventions to prevent the onset of diseases and enhance the accuracy of medical treatments [2]. Moreover, on a broader scale, tracking daily behaviours facilitates lifestyle monitoring, paving the way for offering personalised health solutions.

Daily behaviour refers to a recurring sequence of human movements, actions, or gestures, and the patterns for these behaviours can provide insights into an individual’s daily routines, habits, and activities [8,9]. Examples of behaviour patterns include sleep/wake cycles, bathroom usages, meal times, and mobility routines [10,11]. Hence, the most common way to study an individual’s behaviour is by monitoring everyday activities. Previously, these activities were assessed manually through interviews and questionnaires, which was time-consuming and prone to errors. Luckily, the introduction of IoT technologies and the widespread use of smartphones and wearable devices have made automated monitoring feasible and accessible and benefit this process by making it more efficient and accurate [12,13]. Many consumers purchase a pedometer or wearable fitness device in order to track their physical activity (PA), often in pursuit of a goal such as increasing cardiovascular strength, losing weight, or improving overall health [14].

The main idea of IoT is to connect anything/everything (e.g., sensors, devices, machines, people, etc.) to gather intelligence from such objects [15,16]. In this way, the IoT devices enable continuous monitoring of individuals and understanding of human behaviour by providing valuable objective data on their activities (possibly in real time) [13,17]. Sensor data are collected passively without human effort, allowing users to forget about the device and continue with their day [18,19]. Meanwhile, using data mining techniques on the collected sensor readings, different types of activities can be recognised and classified based on their unique sensor signatures, and in this way, behaviour patterns can be extracted.

The activities that have been explored in the literature are different from the perspective of their impact on health status and difficulty of sensing, detecting, and performing [8,20]. Therefore, different systems consisting of wearable and ambient devices equipped with various types of sensors are commonly used to identify activities and behavioural patterns. Caregivers and researchers are primarily interested in monitoring the ability of individuals to perform two sets of activity classes: activities of daily living (ADLs) and instrumental ADLs (iADLs), because of their role in health management and the assessment of individuals’ ability for living independently [21]. ADLs involve self-care tasks like bathing, eating, walking, and sitting, while iADLs involve activities related to interacting with the physical and social environment, such as food preparation, housekeeping, using the telephone, and managing medications [12].

However, merely detecting the activities and visualising the behaviour patterns is not enough, and individuals need complementary guidance and interpretation from health professionals based on the data and monitoring results. Comparing behavioural routines with desired guidelines to describe how, when, and for how long different activities should or should not be carried out helps in achieving personalised healthcare and self-tracking of adherence to a healthy lifestyle. Therefore, if IoT devices can offer comprehensive and accurate insights into human activities and behaviours, health experts can intervene and provide individualised health services. In this way, accurate recognition of activities and an in-depth and comprehensive picture of behaviour patterns are essential for monitoring and analysing behaviours [22,23]. Achieving this goal using sensors in off-the-shelf IoT devices is a significant challenge for IoT systems in the healthcare domain.

The main problem is that raw data from IoT devices often have inadequate quality, and each sensor type comes with limitations in detecting the activities, which can hinder the full utilisation of IoT systems and data mining potential [24]. As a result, domain experts, such as medical staff, may not fully rely on insights generated by IoT technology and its corresponding analysis and may question the credibility of these systems [25]. Moreover, the outcome of the analysis, e.g., behaviour models, can be hard to comprehend due to their complexity.

This has motivated us to address the problem of using an IoT-based system for behavioural analysis. We aim to explore how understanding daily behaviour routines can assist healthcare providers in their assessment. In our research, we identify daily activities to model several routines for daily behaviour and then investigate how these routines align with health guidelines. We have described our proposed approach for longitudinal monitoring by considering elderly care daily behaviour and lifestyle monitoring in terms of having adequate physical activity levels. We use an IoT system with several sensors to obtain a comprehensive view of human behaviours to achieve this. In addition, we utilise process mining (PM) techniques to highlight routine variations in a clear and understandable way for clinical staff.

Indeed, we aim to investigate how using multiple sources of data and the synergy of information from multiple data sources affects the quality and comprehensiveness of behavioural modelling. We want to determine whether this approach increases the readability and effectiveness of discovered behaviour models or adds to the complexity of the models and makes them unreadable. Thus, to address the need for continuous monitoring of behaviour routines and lifestyle, our paper contributes to:

- **Integrating multimodal IoT data sources into a unified event log:** We combine ambient sensors, smartphone usage, and wearable data to demonstrate the complementary impact of using multiple input sources and the synergy of information for behaviour modelling. Unlike prior studies that often rely on single-modality logs, this integration enables a more comprehensive and reliable view of daily routines and their variations.
- **Employing process mining techniques with a focus on interpretability:** We generate behaviour models enriched with readability-oriented visualisations, including calendar views with zoom levels, timelines with alert signs, and heatmap-enhanced TPAs. These visual tools move beyond conventional PM outputs to facilitate behaviour analysis and make the results more understandable for healthcare professionals.
- **Discriminating daily behaviours based on alignment with health guidelines:** By linking behaviour routines to guideline-based measures (e.g., daily step recommendations), we show how the proposed approach can highlight the reasons for different physical activity levels and provide actionable insights for supporting personalised healthcare.
- **Demonstrating the approach in a real-life elderly care case study with contextual events:** Using a longitudinal dataset that includes contextual external events (COVID-19 lockdown and Ramadan), we address the health-related issue of physical inactivity. The case study illustrates how the framework can reveal behaviour changes linked to external conditions that are both clinically relevant and practically useful for health monitoring.

Compared with existing multimodal IoT research in elderly care, our approach places particular emphasis on improving the interpretability and usability of behavioural insights for health professionals. By combining multi-source data with process mining techniques and visual tools, we not only model routines but also provide actionable, guideline-based insights that can support clinical decision-making. The framework is not intended to replace clinical judgement, but rather to act as a decision support tool: by linking daily routines to health guidelines and presenting them through interpretable models and visualisations, it enables healthcare professionals to assess adherence to healthy behaviours, identify deviations, and plan tailored interventions.

The rest of the paper is organised as follows. In Section 2, some required background knowledge and related works are mentioned. Section 3 introduces the proposed approach and selected elderly care case study with detailed information on the used dataset. Then, in Section 4, we illustrated our experiments on the use case dataset, and the results for investigation of daily routines based on location and activities are represented. A discussion on behavioural analysis is also provided in Section 5. Finally, Section 6 concludes the paper.

## 2. Background

In recent years, many efforts have been made to develop IoT systems in the healthcare domain and to study human behaviours. In this section, the required background knowledge for understanding these systems and a summary of conducted research based on the type of sensors that are used in the IoT system are provided. Moreover, since PM is the main idea and focus of this paper, the IoT systems which use this method for behaviour analysis are briefly reviewed in Section 2.2.

### 2.1. IoT-Based Behaviour Analysis

With the advent of ambient and wearable sensors, many efforts have been made by the academia and industry to leverage IoT and human-centric technologies such as ambient assisted living (AAL), smart homes, and wearables in the healthcare domain in order to address the increasing demand for higher quality of health and well-being services [15,16,17,26]. These systems primarily target elderlies, patients with chronic diseases, and individuals with disabilities to improve their quality of life and independence while reducing the burden on caregivers and healthcare systems. Some examples include fall detection, medication reminders, home automation, and social interaction platforms [27].

In many studies, IoT devices with built-in sensors have been used with machine learning algorithms for continuous long-term monitoring, which refers to collecting data of user’s activities on an ongoing basis without interruption or gaps in data collection [10]. Based on the collected data, the behaviour patterns (or markers) such as sleep/wake behaviours, activities, or activity levels can be identified. These markers were used to predict pain [28] or mobility, cognition, and depression symptoms in older adults [6]. Additionally, the changes in behaviour patterns may also be indicative of health events or changes in health status and provide insights into how individuals with chronic health conditions manage their health [10,29]. Also, the detection of changes in behaviour, resulting from treatment regimens for chronic conditions, could determine prescribed treatment regimen adherence and impact [30]. In the following sections, more detailed information on the benefits of using different sensors in IoT systems for behaviour analysis (Section 2.1.1) and a summary of the proposed works (Section 2.1.2 and Section 2.1.3) is provided.

#### 2.1.1. Sensor Modalities in IoT Systems

As mentioned, multiple sensor types have been utilised in IoT systems for behaviour monitoring so far. Ambient sensors are designed to collect data about the environment and activities within a space without requiring direct interaction or intervention from individuals. They are typically embedded or installed in everyday settings (i.e., homes or buildings) and can monitor various aspects of the environment, including motion, light, temperature, and door usage. On the other hand, wearable sensors are worn by users on the body or clothes and require individuals to wear or carry them [10]. Accelerometers, magnetometers, gyroscopes, or wearable cameras besides devices to measure physiological variables like heart rate and blood pressure are examples of wearables that are used in the existing works [13,17,20].

Ambient sensors operate passively and unobtrusively, allowing continuous and long-term data collection, while wearable sensors can track activities regardless of user location [7]. Moreover, both wearable and ambient sensors have their limitations. Ambient sensors work in a limited area, and using a system with pure ambient sensors is less capable of identifying detailed changes and elaborate actions. Another limitation is that they must be well placed in the environment to record data regularly and accurately [11,31]. When the conditions are not suitable for these sensors, or when they are not installed correctly, the collected data will be incomplete or incorrect [24]. On the other hand, wearable-based solutions also come with the complexity of sensor placements, higher cost, and obtrusiveness for users [32]. Also, wearables rely on battery resources and need to be correctly positioned on the body. If these two things are not performed correctly, wearable-based data collection will be disrupted.

Therefore, each type of sensor has its limitations when used separately. Consequently, the challenges that both sensor types have lay the foundation for developing hybrid sensory systems to tackle these problems [13]. By combining data from multiple sources in healthcare, professionals can make more informed decisions and improve diagnostic accuracy [3]. However, utilising multiple sensors for monitoring massively increases the quantity of available data for analysis, bringing a new complexity level. Regardless of the challenges for storage management, the variety and volume of data complicate the analysis process as it takes time to select the right sensor to detect a specific activity [33].

#### 2.1.2. IoT Systems with a Single Type of Sensor

Ambient sensors have the potential to provide new insights into how individuals such as patients with chronic health conditions manage their health at home. They are used to monitor the behaviour patterns, daily routines, and activities of individuals within the home areas to support timely interventions for optimal health outcomes. For example, movements, sleep interruptions, bathroom usage, and time spent out of the home are listed as behaviour markers in [10]. In this study, these behaviour markers and their descriptive statistics were calculated using sensors in participants’ homes to aid in recognising falls, bowel issues, urinary tract infections, and disrupted sleep. The results represent pieces of evidence that continuous sensor-based monitoring of patient behaviour in home settings can use to provide automated detection of health events, the events that may be difficult to detect through traditional clinical assessments [10].

In another study by Lago et al. [34], behaviour patterns were divided into three types; temporal, location, and frequent activity sets. This study highlights that inferred patterns can change over time due to various factors such as health conditions, new activities, and personal interests. Hence, a one-size-fits-all approach may not be effective, and personalised classifiers capturing the changing behaviour patterns and timely correlations (e.g., weekly, monthly) are recommended [34].

Moreover, wearable devices are also used to provide insights into different aspects of individuals’ daily lives. An investigational method combining physiological and location data gleaned from a wristband is used in [35] to identify daily activities that participants struggled with, providing a more comprehensive understanding of the participants’ experiences. Rodrigues et al. in [36] present a review of techniques based on IoT for healthcare, based on the most recent publications and industrial products available in the market.

#### 2.1.3. Multimodal IoT Systems

As mentioned, to overcome the challenges of different sensors and due to accuracy issues, in some health applications, systems with multi-sensor modalities have been utilised. Various data fusion methods and their applications in different areas have been proposed and reviewed so far. Pires et al. in [27] built a roadmap for achieving the identification of ADLs such as walking, sitting, and sleeping by combining data from accelerometers, gyroscopes, and magnetometers on mobile devices. In addition, a combination of RFID devices for tagging key objects and a single accelerometer on the dominant wrist was used for accurate activity recognition [12].

Also, in a study by Garcia et al. [7], the authors show that if different types of sensors are deployed together in a well-designed and precise manner, the system can provide better accuracy for ADL recognition and behaviour monitoring than if these sensors were utilised separately. They utilised a combination of contact sensors, thermal sensors, and accelerometers. Contact sensors were attached to objects such as doors, cups, kettles, cupboards, and containers, while thermal sensors were used to collect ambient sensor data and accelerometers were used as wearable sensors.

In 2011, EMUTEM (Environment Multimodal pour la Télésurveillance Médicale) was proposed as a multimodal system for in-home healthcare monitoring [37]. It integrates various sensors, including microphones, a wearable device (RFpat), infrared sensors, and home automation sensors, to collect elderly physiological and behavioural data, acoustic environment information, and environmental conditions. Then, each modality’s data is analysed using specific algorithms, and a fusion approach based on fuzzy logic and medical recommendations are employed to combine the outputs from different subsystems. This multimodal fusion enhances the system’s reliability by detecting distress situations and offers a powerful tool for in-home healthcare monitoring.

Crispim-Junior et al. proposed a multi-sensor system to diagnose early-stage Alzheimer’s patients [33]. This multi-sensor surveillance system integrates video recordings and accelerometer data to identify activities within a medical clinical protocol, specifically focusing on iADLs. The system aims to assess the independence, executive functions, and cognitive abilities of older individuals, particularly in relation to Alzheimer’s disease. The inclusion of a wearable accelerometer improved event detection performance and enhanced the accuracy and effectiveness of activity recognition compared with a system relying solely on video data.

In a work by Wang et al. [32], wearable sensors were used to identify specific daily activities and ambient sensors were used to show the user’s daily routine at a room level. In fact, the whole task of recognising all defined activities was divided into several sub-tasks according to the room-level location information captured by infrared sensors. In this way, the computational time drops for each room-level sub-task and the recognition accuracy improves due to avoidance of misclassification for some confusing activities by separating them into different room groups.

In a study by Z. He et al. [38], an elderly care system was proposed, using video processing technology as the core, combined with sound, infrared, and pulse detections. The system aimed to detect abnormal activities, such as falling or staying too long in the toilet, and notify their relatives in case of any unexpected occurrence. Additionally, Cook et al. in [22] utilised ambient and wearable sensors to collect data and extract a set of behaviour markers on a daily basis. This research is associated with the CASAS project and its smart home systems that use binary sensors for behaviour modelling and health monitoring. The study examined behaviour markers to determine whether they contain insights into an individual’s daily routines. The findings indicate a significant connection between these behaviour markers and health status, suggesting that they can be used to track and predict changes in health over time.

### 2.2. Behaviour Process Mining

Typical pattern recognition algorithms use computational techniques to identify common patterns in datasets, leading to mathematical representations or models. While these algorithms have achieved high accuracy in modelling human behaviour, the complexity of the models often makes them difficult for experts to understand and refine. A crucial aspect of modelling human behaviour and habits is the readability and interpretability of the resulting models, which directly impacts an expert’s ability to validate the model [39]. Understandable data-driven systems are therefore preferred, as they allow experts to grasp the reasoning behind the system’s recommendations and provide clues for better decisions in daily practice.

Process mining is a discipline that aims to discover, monitor, and improve real processes by extracting knowledge from event logs (an event log is a set of events within a time interval with every single event occurring at a given point in time [40,41]). Its key goal is to transform event data into actionable insights, using discovery algorithms that prioritise model readability and interpretability. A process is an ordered series of activities that are executed (repetitively) with the aim of achieving a specific goal. Thus, the notion of the process can be leveraged to describe most of the behaviours we adopt in our daily life [42]. As such, PM techniques are increasingly applied to human behaviour modelling, where they allow for the creation of graphical models based on sensor logs—enabling the discovery of structured, understandable representations of human daily routines [2].

In addition to discovery, PM also enables conformance checking, which allows for comparing observed behaviours against reference models or guidelines. This can help assess individuals’ daily routines in satisfying a particular goal, discover the deviations in doing the activities, and even verify the possible ignorance of specific steps [43]. Real-time PM-based monitoring frameworks, such as Viola [44], extend this capability to streaming data by enabling online detection of behavioural conformity.

However, applying PM to raw sensor data presents several challenges. Sensor data typically exists at a much lower abstraction level than process-level activities [45,46]. As a result, bridging the abstraction gap between low-level sensor events and high-level behaviour models is a necessary step for meaningful PM applications. To address this, a wide range of techniques have been proposed, including complex event processing (CEP) [47], LLM-based log abstraction and integration [48], clustering-based activity segmentation [45], and community detection [49]. For example, de Leoni and Pellattiero [50] emphasise the importance of aggregating IoT data to the right granularity for PM, while Weyers et al. [46] introduce “activity signatures” to capture recurring event patterns from sensor streams. These efforts collectively represent the growing body of research on adapting PM techniques to IoT environments and contribute to the broader domain of complex event processing [47].

In line with this, a number of PM discovery algorithms have been adapted to create behaviour processes, as reviewed in a study by Ma’arif et al. [2]. The heuristic miner [51] and fuzzy miner [39] are two popular techniques that simplify process models by removing infrequent transitions, thereby improving their readability. PALIA (parallel activity log inference algorithm) [52] is another discovery algorithm that utilises syntactical pattern recognition techniques to derive expressive workflow models known as TPAs (timed parallel automatons) [53]. TPAs are formal representations capable of encoding parallelism, frequency, and temporal patterns and are known for their low grammatical complexity while maintaining high expressiveness. Unlike traditional PM algorithms, which often overlook infrequent behaviours, PALIA retains rare but potentially meaningful events—such as missing a medication dose that occurs once a week—making it particularly suitable for behavioural analysis and elderly care applications [43].

PALIA has been successfully used in diverse healthcare contexts, such as follow-up protocols of patients with diabetes [54] and behaviour modelling within elderly care scenarios [43]. In a study by Lull et al. in [55], an interactive PM system using PALIA was applied to passive infrared (PIR) sensor data from a solo-resident home. The modelled TPAs enabled analysis of intra-subject variability and detection of behaviour changes through daily process comparisons.

While previous studies have shown the utility of PM techniques in interpreting human behaviour from IoT sensor data [2], they typically focus on single-modality event logs or assume structured sensor data. Recent works have introduced abstraction and fusion methods, but the applicability of PM to truly multimodal datasets that merge low-level, heterogeneous sensor data streams into a unified behavioural process model remains underexplored. Moreover, most existing approaches produce complex models that are difficult for clinicians to interpret in practice.

In contrast, this work expands on existing foundations by proposing a pipeline that combines insights across multiple sensor modalities, applies PM to discover behavioural routines, and enhances interpretability through innovative visualisations. This enables not only the detection of routine variations but also the communication of clinically meaningful insights to healthcare professionals, as demonstrated in our proof-of-concept case study focused on elderly care.

## 3. Behaviour Analysis and the Elderly Health Monitoring Case Study

Our proposed approach aims to tackle the issue of using IoT systems for behavioural analysis. It involves using well-known health measures to group daily data into several days, with labels representing alignment with health guidelines, (e.g., healthy and unhealthy labels). We then examine whether different behaviour routines support achieving health goals and how closely they align with established guidelines. The behaviour routines are modelled and extracted based on a subject’s daily activities and movements, making them highly representative of that person’s habits and characteristics.

The design of the approach explicitly targets healthcare professionals as end users. The models and visualisations are intended to support decision-making by highlighting behaviour patterns that align with or deviate from recommended health guidelines, thereby facilitating the assessment of patient lifestyles and informing possible interventions.

It is important to note that daily routines can change over time due to changes in behaviour (intentionally or unconsciously), health-related issues, or external factors and occasions. These changes in behaviour result in different variants in behaviour models, which can be challenging to identify if the changes are insignificant. In this section, we first provide an overview of our proposed approach and then describe the specifics of the behaviour monitoring case study that we have chosen for evaluation.

### 3.1. The Proposed Behaviour Analysis Approach

**Figure 1:**
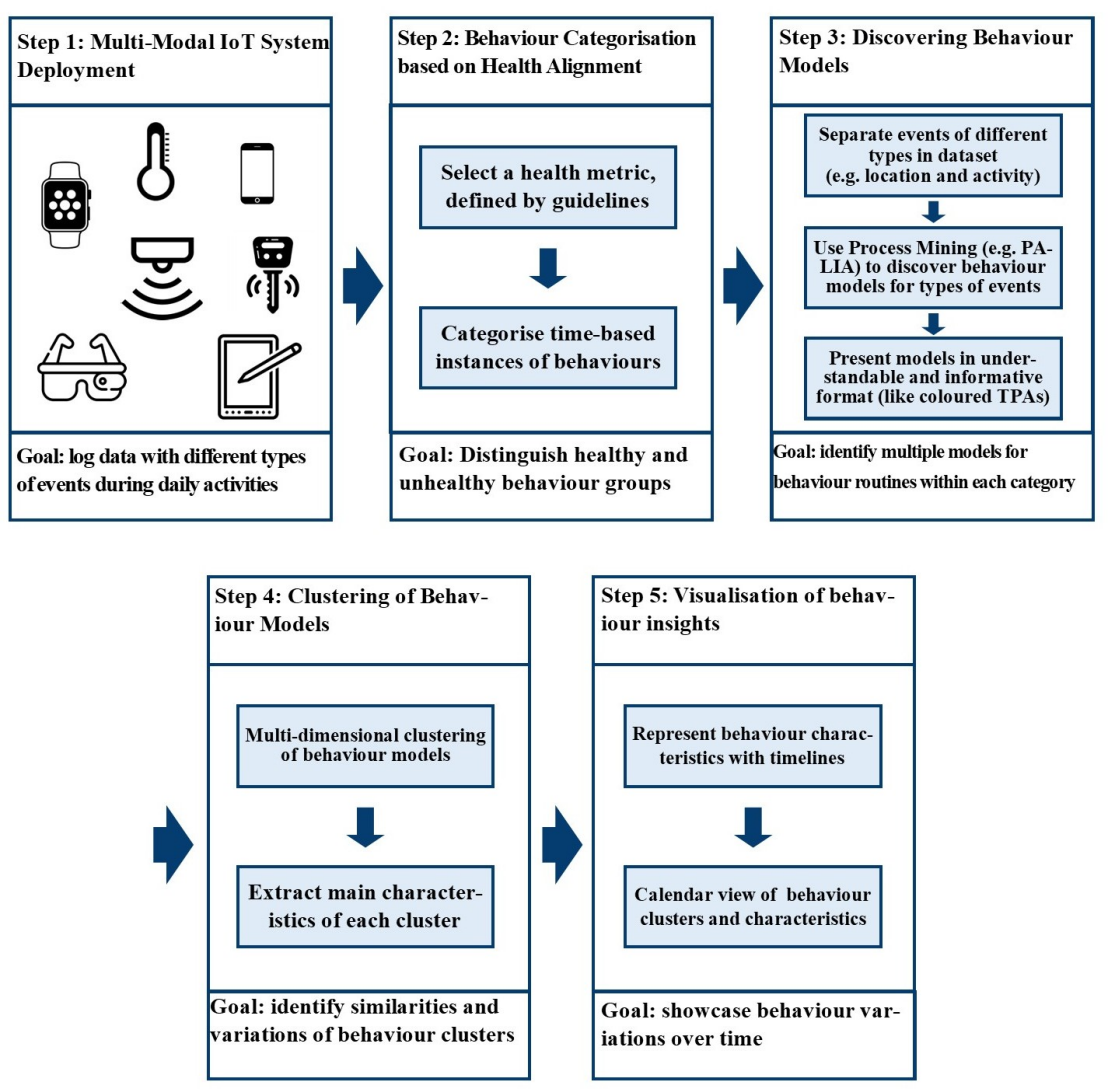
The schematic view of the proposed five-step approach, from multimodal IoT data collection to behaviour visualisation and analysis.

The proposed behaviour analysis approach aims to explore how understanding behaviour routines and using IoT-based systems can assist healthcare providers in their assessment. A schematic representation of it is presented in Figure 1.

To gather information about individuals and their surroundings during daily activities that possibly affect behaviour routines, our **first step** involves deploying an IoT system equipped with various sensors and devices. This system should be designed to be multimodal, tapping into different sources to provide a more complete understanding of an individual’s actions and their environment. Picture it like having a team of sensors, each offering a unique perspective on what is happening. By aggregating these diverse viewpoints and leveraging the synergy of information, we create a holistic and thorough picture of the person’s behaviour and surroundings. This comprehensive understanding serves as a solid foundation for our behaviour analysis.

It is important to note that our framework does not aim to develop an activity recognition algorithm. Instead, we assume that raw sensor events can be abstracted into basic activities and contexts (e.g., using TV, presence in the kitchen) through event abstraction methods, as commonly performed in smart home studies. These activities are then treated as building blocks of daily routines. Our analysis focuses on modelling and comparing these routines over time, linking them to health guidelines, and identifying deviations that may indicate lifestyle or health-related changes.

The **second step** involves using a specific measure as a marker to distinguish between healthy and less healthy behaviours based on their alignment with health guidelines. To achieve this, we establish distinct groups based on how well behaviours align with these guidelines. We categorise behaviours into different groups—one (or more) for those that meet the guidelines and other groups for those that deviate, based on their distance from the expected healthy pattern. We then apply this categorisation to time-based instances of behaviour routines, such as daily or weekly periods, according to the health measure values available in our data. It is essential to choose health indicators that are regularly monitored, as this allows us to consistently categorise behaviour routines. For example, with daily measurements, we can evaluate daily routines, while weekly measurements enable us to assess weekly behaviour patterns. By systematically comparing and studying behaviour routines over different periods, we gain insights into how each period of routines influences health outcomes.

**Thirdly**, we discover behaviour models for each set of behaviour routine instances, categorised based on the defined health measures. We consider all of the traces and their corresponding behaviour models in each category to better understand the patterns within that group. This step involves looking at different types of events recorded by our system, like the location of the person and the activity they are engaged in at a given time, and linking them together based on their time correlation. In fact, multiple event types can describe a single behaviour, which assists us in acquiring multiple models for each behaviour routine, highlighting more information to create comprehensive behaviour models. This approach lets us consider the diverse facets of a person’s behaviour in an interconnected way. By leveraging PM techniques, particularly the PALIA algorithm, we create easy-to-understand models in the form of TPAs. These TPAs, equipped with heatmaps representing duration, reveal differences between routines in a clear and understandable manner for healthcare professionals.

**Next**, when we discovered the behaviour models for each group, we conducted multi-dimensional clustering. In this step, clustering comes into play to identify similarities within each health-related group, aiming to uncover common patterns and dominant events that define a particular class more accurately. Clustering is a classic and widely used pattern recognition technique that enables the grouping of traces (or their corresponding process models) based on structural and behavioural similarity [56]. Importantly, considering the entire set of traces at once can often result in highly complex, spaghetti-like process models that are difficult to interpret [57]. To overcome this, we cluster the traces so that each cluster represents a more coherent set of behaviour patterns, which can be effectively summarised using a single process model.

In behaviour analysis, the duration and frequency of events within discovered models shape the meaning of processes, influencing the corresponding behaviours and the variations. By clustering behaviour models and closely examining the model of each cluster, we can reveal commonalities in behavioural routines and gain insights into which activities contribute to variations, as well as which variations align more closely with physical health guidelines. This approach provides a detailed understanding of the differences in behaviours, guiding us in pinpointing the elements that are most beneficial for health.

**Ultimately**, it is essential to present the outcomes of data analysis in a clear and understandable format for further review by experts. The insights extracted from data only become valuable when they can be easily comprehended and utilised for informed decision-making without requiring extensive data knowledge. To achieve this goal, our proposed approach prioritises visual clarity by opting for PM techniques known for their effectiveness in communicating findings straightforwardly. Our contribution extends to this visualisation aspect by adopting iconic representations of behaviours instead of relying on numerical data; colours are used to indicate durations for easier interpretation. Since we are exploring behaviour variations, presenting these differences over time through a calendar view and timelines of the behaviour instances proves to be an effective solution. These user-friendly illustrations represent the main characteristics of behaviour classes and changes in patterns over time. It acts as a powerful aid for healthcare staff by aggregating all the insights.

### 3.2. Elderly Care Behavioural Monitoring Case Study

To investigate the feasibility and validate the effectiveness of our proposed approach in discovering insights, we conducted an in-depth case study to analyse behavioural routines and highlight variations in daily activities. We utilised an IoT dataset, the DAMMI dataset [58], which captured the behaviour of a 60-year-old woman living independently in her apartment. The data has been collected using ambient sensors installed throughout the house, as well as a wristband and a smartphone.

#### 3.2.1. Data Collection

The sensors in the house are used to perceive the environment, e.g., the activities performed by the resident, during the day. The installed devices are 15 binary sensors positioned on furnishing elements, appliances, and doors. Indeed, the ambient sensory information includes readings from multiple PIR sensors showing the presence of the subject in different areas of the solo-resident house and some activities performed in these areas (like praying or using telephone), a power usage sensor indicating TV usage, contact sensors highlighting the opening and closing of the bathroom, WC, and closet doors, and a gas detection sensor detecting cooking activity. In addition, with the aid of a mobile application, smartphone usage information is collected to show the timestamps of mobile usage and the names of the used applications. Also, the subject wore a wristband (Xiaomi Mi Band 3) to track daily walking steps during data collection.

All data modalities were collected for 146 days between 9 January 2020 and 2 June 2020. During this time, several exciting events occurred, which are likely to have influenced the resident’s behaviour. On the 40th day, the COVID-19 pandemic started, and the region went into lockdown. Additionally, from the 109th day to the 137th day, it was the month of Ramadan, a religious month for Muslims. During this time, the resident likely changed her habits, such as meal times, praying times and duration, and sleeping intervals, due to fasting from sunrise to sunset.

#### 3.2.2. Dataset Pre-Processing and Event Log

The ambient sensors have collected data on the user’s daily activities and locations in the form of a sensor log. Each event in this log represents a measurement taken by a sensor and includes the record’s timestamp, the sensor’s name, and its value (i.e., the measurement or on/off state). The dataset encompasses sensors’ raw readings at a low level of abstraction, representing the presence of the subject near appliances, the open or closed state of doors, and the use of the stove. Since the goal is to monitor the user’s behaviour in terms of daily activities, the dataset needs to be abstracted. To accomplish this, we applied a set of simple rules to the records from PIR and contact sensors to infer the user’s presence in different areas of the home, as well as the time spent there. Each sensor was mapped to its specific installation location. For instance, a record triggered by a sensor in the bedroom closet indicated the user’s presence in the bedroom, while a sensor associated with kitchen appliances indicated both their presence in the kitchen and the performance of related activities, such as cooking. By analysing sequences of sensor activations and their timestamps, we derived continuous events, with duration calculated as the time from the first sensor activation in a location or for a specific activity until movement was detected in another area or a different activity start. In this manner, the raw records were abstracted into location-based and activity-related events for the entire dataset period, which were subsequently used to create an event log. This event log features records that include Case ID, activity name, start timestamp, and end timestamp, suitable for further process mining analysis.

After creating the abstracted events, we performed error correction to handle missing records and noise. Specifically, we applied the hybrid error-correction framework proposed in [24], which combines rule-based and PM-based correction methods. In this approach, errors are first identified by analysing invalid travelled paths and inconsistent sequences of visited areas, considering the intrinsic specifications of the sensors (e.g., sensing range, sampling rate). Rule-based checks are then applied to highlight and correct likely errors based on these patterns. The cleaned log is further processed using an interactive PM-based correction technique, which validates event sequences against a reference model of the house layout and corrects deviations by enforcing admissible transitions. This combined rule-based and PM-based strategy enables reconstruction of missing events, filtering of noisy or duplicated records, and overall improvement in the completeness and reliability of the event log for subsequent analysis.

Furthermore, the mobile usage events from the smartphone log were also inserted into this event log, with events labelled as “UsingMobile” to treat all mobile usage events as similar activities. We then added daily steps data from the wristband to create a single multimodal IoT dataset after cross-checking the events. At the end, the event log includes location events from seven different locations: Bedroom, Bathroom, WC, Corridor, LivingRoom, Kitchen and Entrance, and the activity events are Eating, Eating and Watching, Chores, Sitting, Sitting and Watching, Using the Telephone, Praying, Sleeping, and Using Mobile.

**Figure 2:**
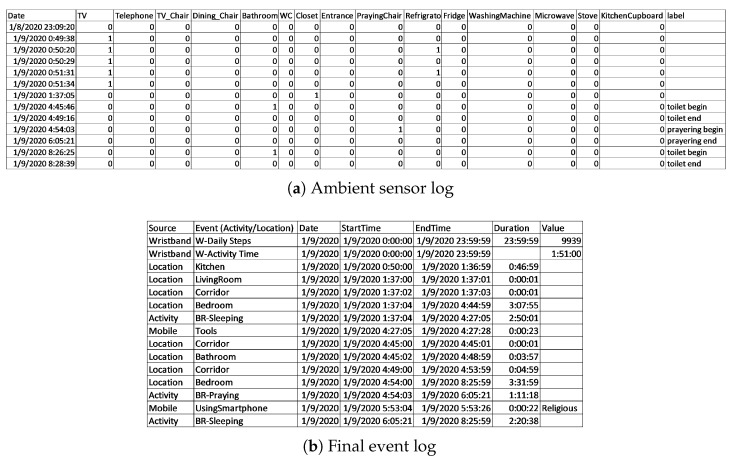
A view of the experimental IoT dataset.

Since we are only analysing a single subject (case) in this scenario, we will examine daily behaviour models by considering dates as Case IDs. This will enable us to discover a process map for each day. It is important to note that we have trimmed the events that started on one day and ended on the next day at midnight and split them into two events. As a result, we have collected events for each day from 00:00:00 to 23:59:59. A part of the sensor log, the extracted location events and the final resulting event log after integration of mobile usage and wristband events are represented in Figure 2.

## 4. Behaviour Analysis Based on Physical Activity Levels

As explained in Section 3.1, in order to implement the proposed approach, it is necessary to group the behaviour routines into separate categories or classes based on their characteristics or a measurable parameter, such as healthy or unhealthy routines. This categorisation enables us to distinguish between the routines belonging to different classes and identify the dissimilarities between them.

Regular physical activity is one of the critical factors in the prevention or treatment of diseases, as well as enhancing overall public health and well-being [19,59]. Thus, an individual’s level of physical activity can serve as a useful indicator of their lifestyle and health condition. Therefore, to show how our proposed approach works, physical activity is used as a measurable parameter to determine different healthy and unhealthy categories, and we aim to explore how daily behaviour routines impact reaching desirable physical activity levels for maintaining good health. To achieve this, we examined the physical activity levels across different days and identified variations in daily behaviour routines that may contribute to these levels. We conducted a two-dimensional clustering to create behaviour models with location and activity information. The following steps outline how we carried out the proposed approach:

1. Define three levels for physical activity: **“Insufficient”**, **“Sufficient”**, and **“Desirable”**.
2. Use a physical activity-based marker (e.g., daily steps) to determine three levels of physical activity and the thresholds for each level.
3. Assign a label to each day within the experiment period based on the thresholds.
4. Group the days with identical labels.
5. Apply a process discovery technique (PALIA) to location and activity events to discover separate process models for each group.
6. Use a clustering technique (QTC algorithm) to cluster the days of each physical activity category based on the structural similarity of their location- and activity-based process models.
7. Analyse process maps of the location and activity clusters to identify the dominant characteristics and behaviours of each group.

By following this approach, we determined what types of activities and daily routines are more effective in increasing or decreasing the daily physical activity level and, therefore, are more useful for physical health.

### 4.1. Defining Levels for Physical Activity

Walking is the most common form of physical activity, and many health initiatives have promoted it as a key strategy for encouraging an active lifestyle. A meta-analysis in [60] has demonstrated a significant inverse relationship between daily step count and both cardiovascular disease and all-cause mortality. With advancements in wearable technology, tracking step count has become increasingly accessible through wristbands, enabling individuals to monitor adherence to such recommendations.

However, the recommended daily step count varies across studies, typically ranging between 4000 and 18,000 steps. Among these, a target of 10,000 steps per day has been widely recognised as a reasonable benchmark for healthy adults [61]. Recent large cohort studies (including [62]) also suggest that benefits begin at around 4000 steps/day, with higher increments providing greater protection against mortality risks.

In our study, we therefore defined two thresholds based on both the literature and the observed distribution of step counts in our dataset. Days with less than 4000 steps were categorised as Insufficient, 4000–10,000 as Sufficient, and higher than 10,000 as Desirable. This categorisation reflects both established guidelines and the variability in our case study, while acknowledging that the ideal thresholds may vary depending on age, weight, and overall health status [60,61]. The daily step count data collected by the wristband is used to determine three distinct physical activity levels:

- **Insufficient Level:** For daily steps less than 4000;
- **Sufficient Level:** For daily steps between 4000 to 10,000; and
- **Desirable Level:** For daily steps more than 10,000.

**Figure 3:**
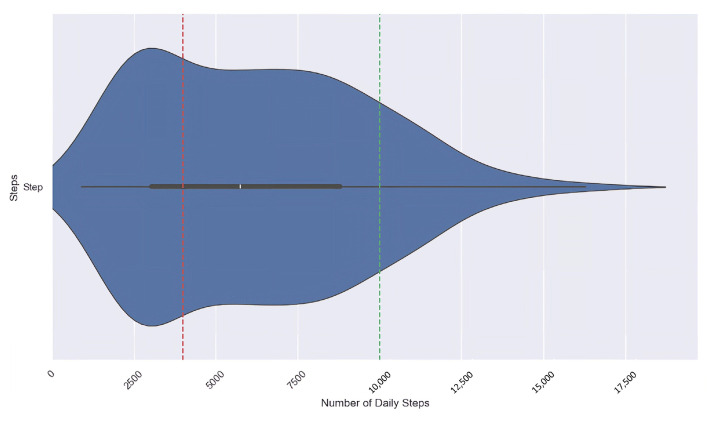
The variation of daily steps in our dataset. The guidelines for specifying physical activity levels are marked with red (4000 daily steps as the threshold between insufficient and sufficient) and green colour (10,000 as the threshold between sufficient and desirable).

Figure 3 illustrates the distribution of daily step counts over 146 days, along with two guidelines representing the thresholds of 4000 and 10,000 for the three physical activity levels. By applying these thresholds, we categorised each day in our dataset according to its corresponding physical activity level, which resulted in three groups: Insufficient (50 days), Sufficient (74 days), and Desirable (22 days), each representing a different level of physical activity.

### 4.2. Discovering Behavioural Routines for Physical Activity Level Groups

As mentioned in Section 2.2, PALIA is a syntactic process discovery algorithm specifically designed to infer workflows from temporal sequences of activities. It generates a readable model of the process in the form of a formal automaton called TPA. PALIA has been successfully applied to indoor locations systems and smart homes to analyse people’s movements and monitor behaviours [43,55]. It provides valuable insight for long-term behaviour monitoring and anomaly detection. In our case study, we have employed PALIA to model the processes of location and activity events for each group.

We then clustered the days in each physical activity level group into multiple clusters based on the similarity of their process models and extracted the main behavioural characteristics of the days within each cluster. By clustering the process models, we are able to group the most similar location/activity behaviour patterns together, and then the models of clusters are used in association with statistical analysis to inspect the possible correlation between behaviour patterns and physical activity levels.

In this regard, the quality threshold clustering (QTC) algorithm is utilised to cluster the discovered behaviour process models. QTC has been used effectively in behavioural analysis literature [55,57] as it allows control over intra-cluster similarity while avoiding the need to predefine the number of clusters, making it well suited for exploratory behaviour analysis. The algorithm requires two parameters: (i) a similarity threshold, which specifies the maximum distance allowed between traces within the same cluster; and (ii) a minimum density threshold, which defines the minimum proportion of traces required to form a valid cluster. If this density requirement is not met, the traces are considered outliers and excluded from clustering [55].

In this study, we explored different parameter settings and selected values that provided meaningful and interpretable clusters while balancing cluster granularity and outlier rates. For location-based process models, similarity and join thresholds of 25% and 5% were chosen, which produced clusters with adequate homogeneity without excessive fragmentation. However, for activity-based clusters, due to the higher variability of activity routines, higher similarity thresholds were applied to partially embrace the inherent fluctuations in behaviour routine and avoid an excessive number of small clusters. These parameter choices were empirically validated by testing multiple configurations and selecting those that yielded the most representative clusters for subsequent behavioural analysis. In the following sections, the results of discovering the location and activity-based process models and their clustering are presented.

#### 4.2.1. Group 1: Insufficient Physical Activity Level Clusters and Main Routines

This group pertains to the 50 days where the subject did not cover the required 4000 steps and her physical activity was not enough. Therefore, we analyse the daily behaviour routines of these days to understand the activities and the areas of the house that the subject spent time on to identify the patterns responsible for the lack of physical activity.

##### Location-Based Clustering

**Figure 4:**
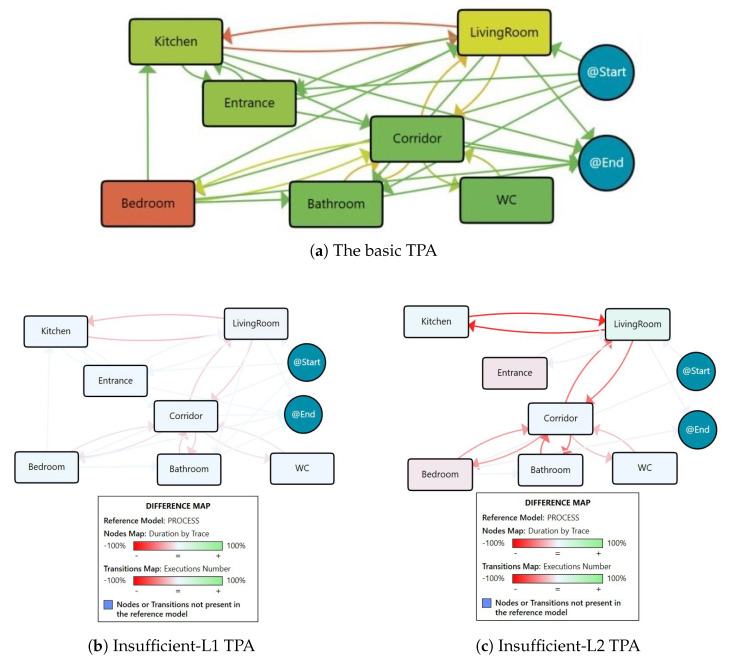
The TPA models for the location-based clusters of days within the insufficient physical activity group. Nodes in each TPA represent different locations of the home, and arrows indicate the transitions.

The process model based on the location events by considering all of the 50 days within the insufficient physical activity group is illustrated in Figure 4a. It is apparent that the person mostly spent her time in the Bedroom (half of the day) and the LivingRoom is the second most stayed place. Additionally, there were many transitions observed between the kitchen and the LivingRoom.

The location-based process maps within the insufficient physical activity level group are clustered into two main groups of 38 days and 6 days, while the other 6 days are ignored and labelled as outliers due to their lack of similarity with other models. The process maps for these clusters are presented in Figure 4b,c, and the primary characteristics and followed routines for each cluster are summarised in Table 1. It should be noted that the nodes and transitions in the cluster maps are coloured relative to the basic location process map.

The difference between process maps and statistics of these two clusters highlights that for the days with insufficient physical activity, we have a pattern related to the Insufficient-L1 cluster, in which the subject spent almost more than 12 h of the day in the Bedroom and about two hours Out-of-Home. On the other side, for the days in the Insufficient-L2 cluster, the behaviour pattern is different and the times for these places decreased, and instead, time spent at the LivingRoom (≥7 h) and Kitchen (≈3.5 h) almost increased by 25% and 15%, respectively. In addition, the transitions between almost all areas of the house are decreased relative to the basic model. Therefore, the proportion of the day that the person went out and was active was small compared with the proportion that they were at home.

##### Activity-Based Clustering

**Figure 5:**
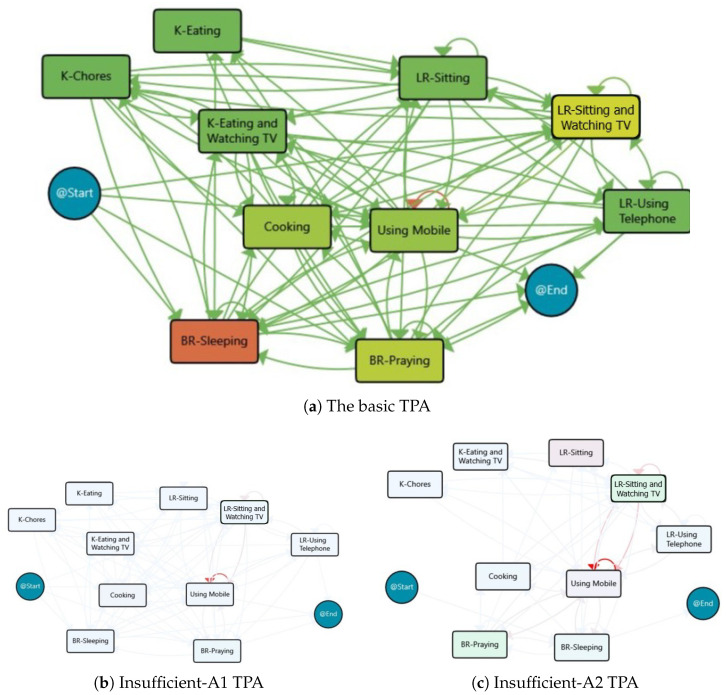
The activity-based TPA models for the clusters of activity routines within the Insufficient physical activity group. Nodes in each TPA represent performed activities in a day, and arrows indicate the transitions.

The process model based on activities for the Insufficient group is also discovered in order to provide insight into the impact of different activities on physical activity. The result is provided in Figure 5, and Sleeping is the most frequent activity in this group.

The clustering based on activity process maps also divided the days with insufficient activity levels into two main clusters (the clustering was based on similarity and join parameters of 35% and 5%, respectively). One cluster had 31 days, another cluster had 6 days, and 13 days remained unclustered. Figure 5 displays the process maps for the derived clusters, and Table 2 outlines the main characteristics of each cluster. Both clusters show that the subject slept for about 8 h and spent approximately 2 h Cooking and 1.5 h using her smartphone. However, the second cluster had more time allocated for Praying and Watching TV.

The location and activity-based clusters clearly highlight that on days that are recognised as days with insufficient physical activity, the subject was mostly inactive throughout the day. She spent the largest portion of the daytime in a sedentary state, engaging in activities like Sleeping and Sitting (for Praying and Watching TV) and only leaving the home for a short period. Additionally, the transitions inside the home were also low, and these routines are aligned with the expectations for the days with insufficient physical activity.

#### 4.2.2. Group 2: Sufficient Physical Activity Level Clusters and Main Routines

The same approach for clustering based on locations and activities is also applied to the 74 days with the label of Sufficient physical activity level, the days with values between 4000 and 10,000 for measured daily steps.

##### Location-Based Clustering

**Figure 6:**
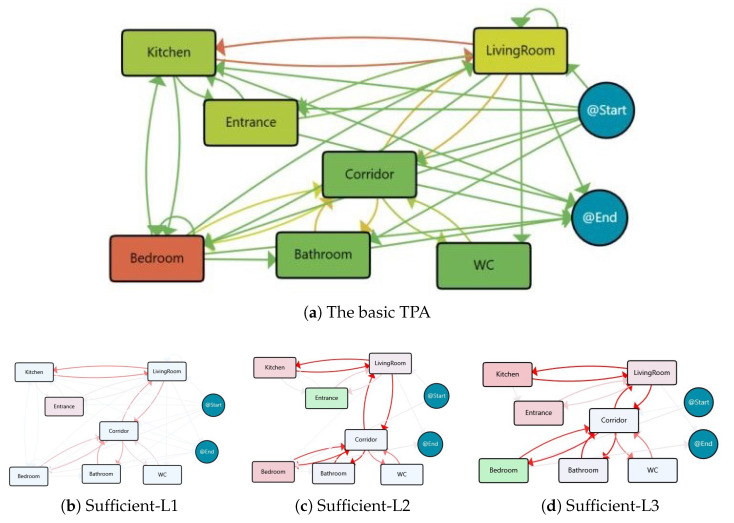
The location-based clustered TPA models for the days within the Sufficient physical activity group.

The extracted location-based process map for the Sufficient group is illustrated in Figure 6a. According to the TPA model, the days within the sufficient physical activity group follow the daily routines where the most time is spent in the Bedroom, LivingRoom, and Entrance (outside of the home and Kitchen, respectively). Similar to the preceding group, frequent transitions between the Kitchen and the Living Room are evident.

The location-based process maps associated with the days exhibiting sufficient physical activity levels are grouped into three primary clusters, containing 53 days, 6 days, and 4 days. Conversely, the remaining 11 days are categorised as outliers. Figure 6b–d show the process maps for these clusters, while Table 3 provides a summary of the key characteristics and routines followed in each cluster.

The differences in daily routines between the clusters in the Sufficient and Insufficient activity groups reveal valuable insights into the subject’s behaviour. Specifically, within the Sufficient-L3 cluster (which includes 4 days), we observed that, on average, the subject spent approximately 15 h in the Bedroom—an unusually high duration compared with all other clusters in both the Sufficient and Insufficient groups. However, despite this extended time spent in a single room, likely engaged in sedentary activities, the subject also recorded around 2.5 h spent outside the home. This outdoor activity likely contributed to reaching at least 4000 steps, leading to the classification of these days within the Sufficient activity group. Thus, the prolonged inactivity period is not reflected in the daily step count, as it is compensated by sufficient activity during other parts of the day.

Conversely, the Sufficient-L2 cluster (which includes 6 days) is characterised by considerable time spent out of the home. While extended Out-of-Home duration often correlates with higher activity levels, these days were still classified as Sufficient rather than Desirable. This suggests that although the subject was outside for extended periods, some of this time may have been spent in low-activity states, leading to moderate overall physical activity levels rather than optimal levels.

Therefore, these examples highlight the importance of multimodal analysis, demonstrating that relying solely on step count or location-based analysis may not always give a complete picture of physical activity levels.

##### Activity-Based Clustering

**Figure 7:**
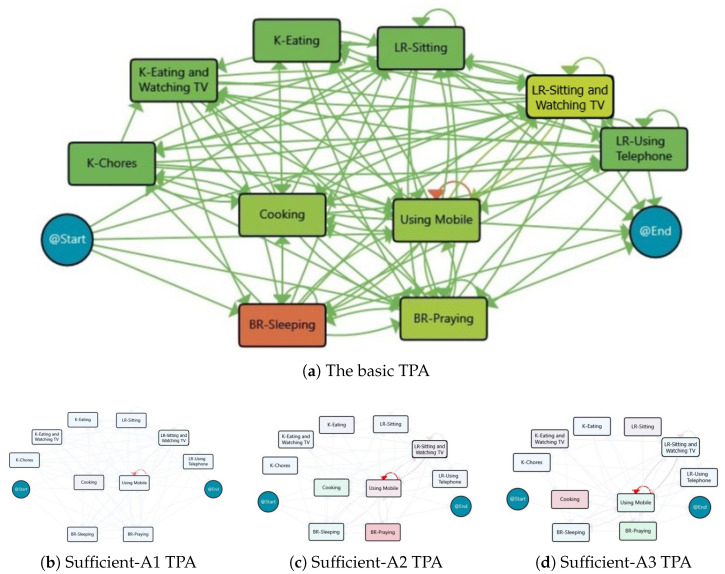
The activity-based TPA models for the clusters of activity routines within the Sufficient physical activity group.

The process model based on activities for Sufficient group is shown in Figure 7a, which indicates that Sleeping is the most frequent activity in this group, just like the Insufficient group. Also, the process maps for the three derived clusters are provided in Figure 7b–d, respectively, and Table 4 summarises the main characteristics of each cluster.

From an activity perspective, the days with sufficient physical activity level are clustered into three classes. The first cluster, Sufficient-A1, consists of 55 days with around 8 h of Sleeping, more than 2 h of Praying, 3 h of Watching TV, and 1.5 h of Cooking and Using Mobile. This pattern is almost repeated for the majority of the days with sufficient activity levels. However, for 8 days in the second cluster, Sufficient-A2, there is an increase in Sleeping and Cooking, but less time is spent on Praying. Oppositely, the third cluster, Sufficient-A3 consists of 5 days belongs to the behaviour models with more Praying and Using Mobile, but less Cooking. Additionally, there are six outlier days that do not fit into any of the three clusters.

#### 4.2.3. Group 3: Desirable Physical Activity Level Clusters and Main Routines

There are 22 days in our dataset that have more than 10,000 covered daily steps, and they are included in the Desirable group. Thus, to highlight the correlation between locations and activities with the high physical activity level of these days, the same analyses based on the proposed approach are applied to the location- and activity-based behaviour models of these days.

##### Location-Based Clustering

The location-based process maps associated with the physical activity level group exhibiting desirable levels are grouped into three primary clusters, containing 15, 2, and 2 days, and the remaining 3 days are categorised as outliers. Figure 6b–d show the process maps for these clusters, while Table 1 provides a summary of the key characteristics and routines followed in each cluster.

**Figure 8:**
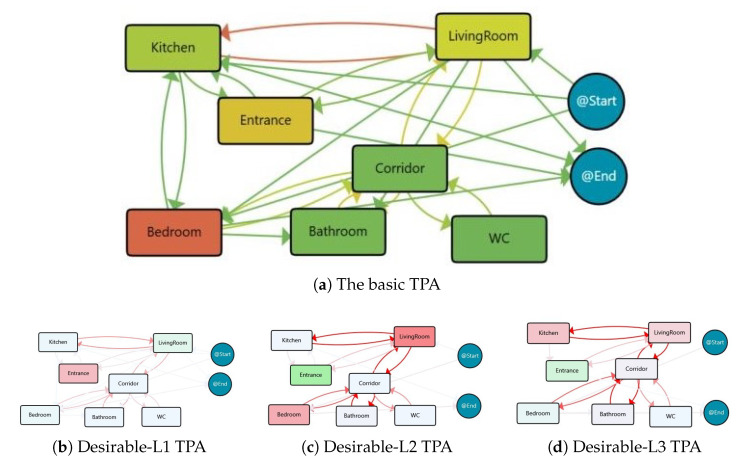
The location-based clustered TPA models for the days within the Desirable physical activity group.

Regarding the 22 days with desirable physical activity levels (i.e., the days that have more than 10,000 daily steps), it is expected that the subject spends more time out of the home. As shown in Figure 8a, the TPA models indicate that the days falling within this category mostly follow daily routines. These days are typically spent for approximately 10 h in the Bedroom and around 6 h outside the home. Similar to the previous group, there are significant movements between the Kitchen and the Living Room.

The desirable physical activity level group’s associated process maps are classified into three primary clusters, which last for 15 days, 2 days, and 2 days, respectively. The residual 3 days are identified as outliers. The process maps for these clusters are displayed in Figure 8b–d, while Table 5 summarises the main features and behaviour followed in each cluster.

The resident for the days in Desirable-L1 cluster spent roughly 5 h outside of the home each day, and this time is even higher for other clusters. By contrast, less time spent in different areas of the home include Bedroom, LivingRoom, and Kitchen in relation to the clusters for other groups. This underscores the importance of leaving the house to engage in physical activity.

##### Activity-Based Clustering

**Figure 9:**
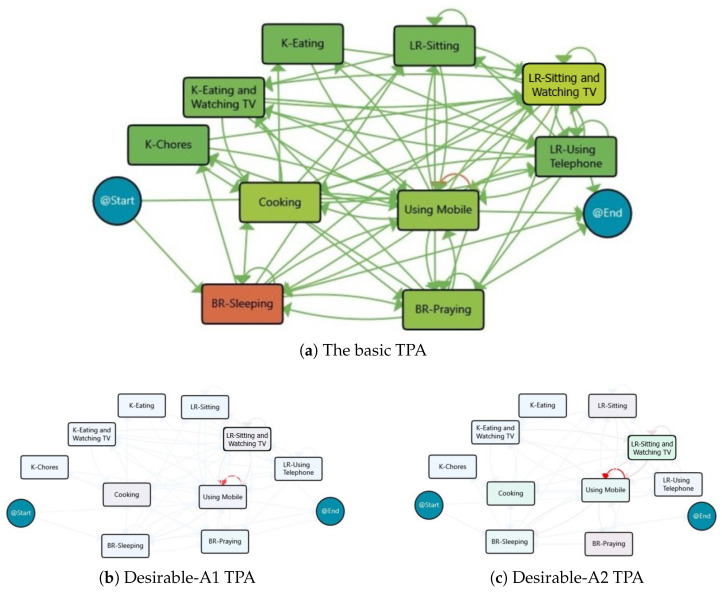
The activity-based TPA models for the days within the Desirable physical activity group.

To examine the impact of activities on achieving desirable physical activity, the process models of days in the Desirable group are discovered by taking into account the activity events. Two classes were identified after clustering analysis—Desirable-A1 with 14 days, Desirable-A2 with 5 days, and 3 days that did not fit into these clusters. The TPAs for the behaviour models based on the activities are illustrated in Figure 9, and Table 6 highlights the main features of the clusters. The results show that Sleeping for about 7.5 h and Watching TV for about 3–4 h are the activities with the highest duration. The main difference between the days in the Desirable-A1 and Desirable-A2 clusters is that the subject spent an additional hour on Watching TV and Cooking on the days in the latter cluster.

### 4.3. Calendar and Timeline View—Looking into the Variations of Behaviour Routines over Time

**Figure 10:**
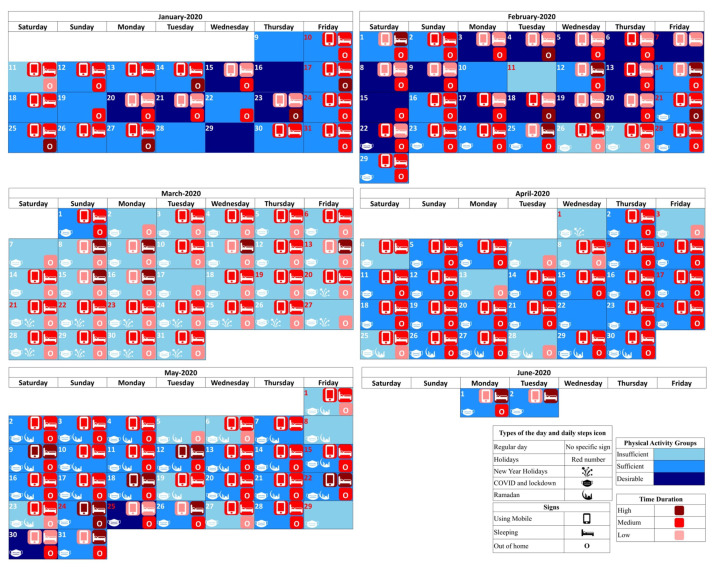
A calendar to visualise all the extracted information in a single view. Daily routines in the calendar view are represented with cells that are coloured based on the physical activity level. In each cell, the icons with mark selected location: Out-of-Home; and activities: Sleeping and Using Mobile.

To effectively summarise the behavioural analysis and investigate variations over time, we propose a refined visualisation approach that includes a calendar view and a series of timeline representations. These visualisations provide a structured and illustrative method for examining behaviour patterns while allowing for different levels of granularity. With the calendar view, all key findings based on the physical activity level analysis and discovered clusters for daily behaviour routines, in association with daily step counts, are visualised in a single unified calendar (Figure 10). Days categorised into Insufficient, Sufficient, and Desirable activity level groups are represented with distinct and contrasting colours (ranging from light blue to dark blue). Additionally, clustering details related to time dedicated to different activities and locations are presented through coloured icons, where darker shades indicate a great duration spent on that specific event for the corresponding day.

Furthermore, the calendar view incorporates contextual markers by displaying icons for external influencers, enabling intuitive exploration of their impact on daily routines. To enhance clarity and reduce complexity, only selected icons based on expert recommendations for key activities are shown in the the high-level calendar view (Using Mobile, Sleeping, and Out-of-Home activities). This summarised calendar view ensures readability while capturing essential behavioural trends.

**Figure 11:**
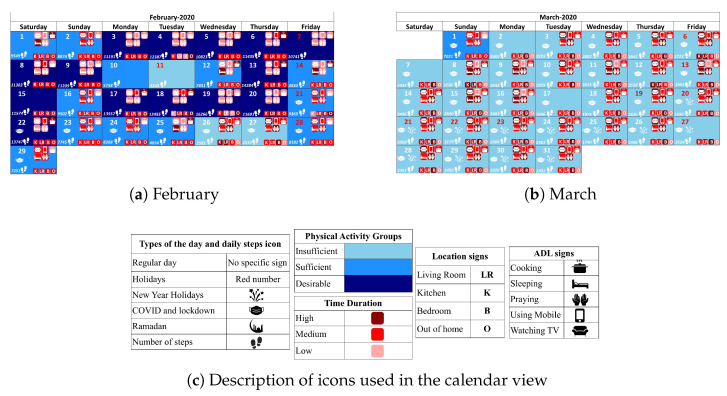
A detailed calendar view for selected months (February and March) to visualise all the extracted information in a single view. With the start of the COVID-19 pandemic and subsequent lockdown in the region (represented with a mask icon in the calendar), the number of days categorised as having desirable physical activity levels has drastically declined.

In addition, to enable multiple levels of granularity, the calendar visualisation tool allows zooming into specific periods for a detailed analysis of behavioural patterns. To illustrate this functionality, we created detailed calendar views for February and March (Figure 11a,b), allowing researchers and health professionals to focus on specific months as needed. The high-level calendar provides a broad summary, while the detailed calendar version offers a comprehensive breakdown of daily activities and behaviour variations. This structure ensures flexibility, allowing users to seamlessly transition between a general overview and more detailed insights, facilitating a balance between clarity and comprehensiveness.

**Figure 12:**
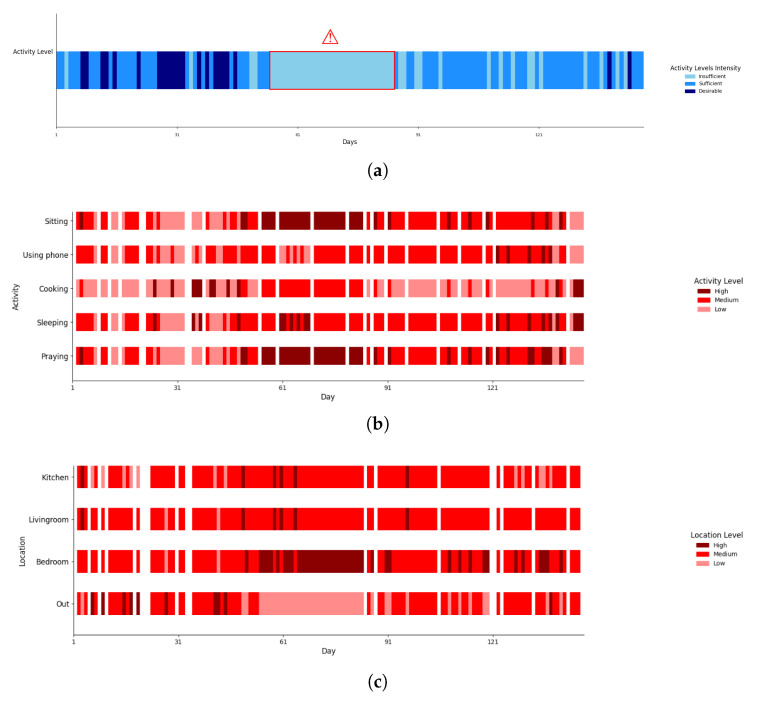
A timeline view to represent variations of (**a**) Timeline of daily activity level over data collection period with an alert sign for more than 7 consecutive days with insufficient activity, (**b**) Timeline of activity events and (**c**) Timeline of location events. The intensity of colours indicates the physical activity group and duration for each location or activity. The red alert sign marks an extended insufficient activity period, which can be linked to spending less time out of home and more time on engaging in sedentary activities in the Bedroom.

To further improve clarity and accessibility, we incorporated timeline plots that illustrate behavioural trends over time in an intuitive and structured manner. These timelines enable a quick assessment of trends by representing each day’s attributes using three shades of a single colour, where darker shades correspond to higher event durations. Daily physical activity levels are plotted in Figure 12a, which highlights periods of inactivity and changes in physical activity trends. Additionally, to support early detection of prolonged inactivity, an alert sign (e.g., a red warning symbol with a border) is displayed when there are extended sequences of insufficient activity (e.g., more than seven consecutive days). The time spent on different activities and in different locations (based on the identified cluster for each day) are illustrated in Figure 12b,c, respectively, over all days of data collection.

Upon examining the calendar and timeline views, and taking into account the start of the COVID-19 pandemic (which began on 20 February) and subsequent lockdown in the region, it is obvious that the number of days categorised as having desirable physical activity levels has drastically declined. Notably, in March, the subject did not reach desirable physical activity levels on any day, with daily values for out-of-home events showing only low to medium durations, marked in light red or red. This suggests the subject stayed at home during the lockdown, as expected. In parallel, the duration spent in the Bedroom has increased, contributing to the decrease in overall physical activity.

A similar change in behaviour routines is expected during the month of Ramadan due to fasting and altered meal schedules. The analysis revealed that during Ramadan, cooking durations were categorised as medium on only five days, while 21 days were classified as low-duration cooking days. In contrast, after Ramadan, there was a significant increase in cooking time, with four out of ten days being classified as high-duration cooking days, compared with only nine days out of one-hundred seventeen before Ramadan.

This has motivated us to also compare the number of days for each physical activity group according to prior-known events, which is reported in Table 7. The results are aligned with the findings from the calendar and timelines.

In summary, the evaluation of this elderly care case study demonstrates the potential of the proposed approach for behavioural analysis in tracking behaviour routines and assessing adherence to health guidelines, such as physical activity recommendations. Continuous data collection via IoT devices and behavioural process model discovery enables the identification of behaviour patterns, making it easier to detect lifestyle changes and deviations from normal routines. The proposed visualisation tools enhance the understanding of behaviour variations over time by combining calendar-based and timeline-based views. This hybrid approach offers a flexible and intuitive method for tracking daily routines and identifying potential concerns, which hold practical value for health practitioners in detecting behaviour shifts early and recommending appropriate interventions when necessary. For instance, it becomes easy to identify days or weeks when a person did not engage in adequate physical activity and to determine the reasons and daily routines that led to this lack of activity. Future extensions of this work could include automated alerts for significant behavioural deviations, ensuring timely responses to critical changes in activity or lifestyle patterns.

## 5. Discussion

Our activities and behaviours have a profound impact on our physical health, forming a crucial connection between daily routines and our health status. In this study, we showcased the potential of conducting behavioural analysis to gain an understandable picture of health conditions. To perform this, we focused on leveraging information from daily life, collected by IoT devices, and proposed a method to extract insightful knowledge on behaviour routines. By inspecting daily routines in terms of locations and activities, we were able to investigate how closely individuals aligned with well-known health guidelines, such as recommended values for daily steps. Our analysis demonstrated that identifying daily routines provides a comprehensive view of individuals’ habits and is a critical foundation for further health investigation. This comprehensive view empowers health experts to understand the actual patterns followed by patients in reality. As a result, they can provide more accurate interventions and enhance the potential for timely and effective health monitoring and treatment.

Beyond the specific findings of this study, the potential of behaviour analysis is not restricted to the example and solution presented in this article. It offers a wide scope for the development of innovative healthcare systems. The interplay between behaviour and health can be explored and investigated from various perspectives that require additional research and inquiry in the future. In the following, we will briefly mention some of these perspectives.

- **In-depth behaviour exploration:** Physical inactivity poses a significant health risk, and while tracking daily steps (as considered in our case study) is a simple measure, it only captures the absence of walking or running activities. However, there are other dimensions to physical activity, such as prolonged periods of remaining stationary or engaging in sedentary behaviours, which can also impact health negatively. Therefore, a comprehensive investigation into extended periods of sedentary routines and the effects of both sedentary and active tasks on overall health is another crucial aspect in discovering the experienced physical inactivity. For instance, as a future work in our case study, we plan to quantify this type of inactivity by accounting the time spent in activities that are conducted while seated, such as watching TV, eating, praying, and using the telephone.
  Besides physical activity, exploring the influence of certain daily life habits on behaviour and health, like the following examples, may also be of interest:
  – Examining sleep patterns, including the time, frequency, and quality of sleep, and their impact on both physical and mental health, as well as subsequent behaviours.
  – Investigating the correlation between dietary habits, calorie intake, and expenditure and their implications for overall health and weight control.
  – Inspecting smartphone usage, along with exploring various mobile-based activities like gaming or social media, and their effects on physical health.
  – Assessing the impact of the work environment on behaviour and comparison of patterns in week and weekend days.
- **Early spotting of behaviour changes:** Gaining a thorough understanding of individuals’ daily patterns and behaviour evolution allows for the timely detection of deviations, particularly valuable in monitoring conditions such as dementia and Alzheimer’s disease. In these cases, labelling daily behaviours as healthy or unhealthy and discovering the clusters that deviate from health guidelines could assist in identifying the main reasons for behaviour changes. Also, visualisation tools like the calendar and timelines can illuminate the onset of changes. Moreover, by harnessing online data collection, these types of behaviour analysis and monitoring approaches support the prompt identification of behavioural shifts, paving the way for early medical interventions that have the potential to impede the progression of diseases. This proactive strategy enhances our ability to address health concerns at an early stage, contributing to improved outcomes and quality of life for individuals affected by these conditions.
- **Longitudinal monitoring and tracking adherence to therapy:** Regularly monitoring the behaviour of patients over time is an effective way to keep track of chronic diseases. This approach facilitates identifying any unusual patterns that may indicate health problems, which is particularly important for conditions such as cardiovascular disease and diabetes. The insights gathered on the behavior patterns can be summarised and presented in daily, weekly, and monthly reports. These reports can be used as a foundation during regular follow-up appointments with health experts to gain an overall view of the patient’s condition and what has transpired between sessions. Instead of relying solely on what patients report, the doctors can refer to actual data to determine if they are taking their medications as advised and figure out how effective the treatment is. This helps them make adjustments to medications, try alternative prescriptions, and ensure patients receive the appropriate care.
  Moreover, behavioural analysis can provide valuable insights into the recovery progress of people after surgery or rehabilitation from a health problem. Early detection of any issues in treatment is critical, and tracking adherence to therapy and recovery progress can facilitate this process. It enables physicians to monitor the health of their patients and provide timely care when needed.
- **Lifestyle Monitoring and staying informed:** Many people today are interested in staying healthy and following routines and diet plans to keep fit. The increasing popularity of wearable devices, smart scales, and health apps that track food and workouts is a testament to the fact that people are interested in being more self-aware about their health. In this situation, a system that utilises a behaviour modelling approach, like the proposed one, can create easy-to-understand reports that visualise daily routines and behaviour. It helps them keep track of their routines and see how well they are following health guidelines. It allows individuals to audit adherence to health guidelines, and then the insights gained can contribute to refining and updating these guidelines based on new discoveries. This system can look at behaviours and lifestyles, considering events and outside factors. If it notices any unhealthy changes, it can alert the user and provide feedback to return to a healthy lifestyle. It is like having a friendly reminder to stay on track and make choices that are good for your health.

The proposed approach has a wide range of applications beyond just health-related perspectives. It is not limited to analysing human behaviour; it also holds significant potential in various other fields. The steps and operations of this approach are versatile and can be adapted for diverse applications, such as analysing consumer behaviour, business processes, or societal trends. By utilising information about processes, routines, and trends and grouping them according to guidelines or preferences, clustering techniques can be applied to uncover patterns and characteristics in any dataset where categorisation guidelines exist. For instance, targeted marketing strategies can be developed by analysing customers’ purchase history, and the productivity and inefficiencies within work environments can be assessed by examining employee behaviours, activities, and interactions. The ability to provide a comprehensive view of trends and visualise the evolution in diverse datasets underscores the universal applicability of behavioural analysis, like the proposed one. As long as guidelines exist to categorise and align data, this approach becomes a valuable tool for extracting meaningful insights and facilitating informed decision-making in various domains.

Beyond exploring behavioural analysis possibilities and use cases, it is crucial to understand the technology that makes these applications work, as it directly impacts the effectiveness of healthcare services. Our case study showcased the power of using IoT systems for data collection and demonstrated how information from various sources gives us a more complete picture for behavioural analysis. We collected daily steps from a wristband, tracked smartphone usage, and observed movements and activities through ambient sensors, allowing us to analyse behaviour based on locations and activity clusters. Moving forward, we can extend this approach by tapping into smartphones to capture social interactions, smart home devices for entertainment data, sleep patterns, and even physiological variables like blood pressure and heart rate through wearables. A multimodal IoT system with diverse data types collected from different devices can bring together much of this information in order to not only provide a thorough understanding of physical activities but also shed light on the context and situations individuals experience, broadening the scope for a more comprehensive analysis.

Building on these possibilities, another important strength lies in the synergy of information from multiple sensors, which creates a stronger and more reliable picture than any single source can provide. Multiple sources of information could complement each other. Our case study demonstrated this synergy by using the data from inside the home (gathered by ambient sensors), combined with the wristband and smartphone info when the person is outside. This combination gives us a more complete view, preventing us from relying too much on just one perspective. For example, we noticed some days (four days in the Sufficient-L3 cluster, “Location-Based Clustering” part in Section 4.2.2) with adequate steps, labelled as days with sufficient physical activity. However, the ambient data showed that during those days, the person spent a long time in the Bedroom, mostly doing sedentary stuff—not so great for health. This problem is something we would not catch if we only looked at the steps and showed the impact of conducting multimodal monitoring. Combining data from different sources helps us spot patterns, like being inactive for a long time and then suddenly being super active, which is not good for health. Our case study and the contribution in aggregating information from various sources clearly showed that it is feasible to achieve a more detailed and reliable analysis. This can reveal the complex dynamics of behaviours and their impact on health.

Ultimately, there is no doubt that innovative approaches for behavioural analysis, like the one proposed in this article, not only enhance the understanding of individual behaviours but also open avenues for personalised healthcare interventions, promoting a healthier and more informed lifestyle. However, these systems and the use of IoT devices for monitoring personal life still need more widespread acceptance. People must embrace the idea of using IoT devices to track their daily lives, while health experts welcome these systems and are willing to use them. This acceptance is the key to making smart healthcare solutions successful and widely used in our society.

However, acceptance alone is not sufficient. Security, privacy, and ethical considerations are critical to ensuring that such systems gain trust and adoption. Continuous monitoring inevitably generates highly sensitive personal data, and protecting this information against unauthorised access and misuse is essential. The widespread acceptance of IoT systems—and the future deployment of frameworks like the one we propose—depends on embedding these safeguards as integral components to guarantee responsible and ethical use of IoT-based monitoring in healthcare. Data must therefore be collected, processed, and analysed with security- and privacy-aware solutions; otherwise, large-scale real-world implementation cannot be expected. Addressing these challenges is a prerequisite to moving from proof-of-concept studies to broader validation and clinical uptake. Finally, adoption of such frameworks in practice requires integration into healthcare workflows and appropriate training for clinicians, which represent non-trivial barriers to real-world deployment.

In addition to these adoption challenges, it is important to note that there are also methodological limitations in the current study. While this study qualitatively demonstrated the added value of integrating multiple modalities, such as revealing prolonged sedentary periods that would not be detected through step counts alone, we did not include a quantitative metric for assessing multimodal gains. This limitation is acknowledged as necessary evaluations require structured comparisons between single-source and multimodal analyses, as well as systematic assessments by healthcare professionals over time.

Additionally, the current evaluation is based on a single case study, which, while limited, is considered acceptable. This case study serves as a proof of concept, highlighting the strengths of our framework in analysing behaviour and identifying changes. Beyond this specific scenario, the proposed pipeline is inherently generalisable and can be applied to any use case where a multimodal IoT system generates an event log. For example, in multi-user contexts, each individual can be represented with a separate case identifier in the event log, allowing for clustering techniques to be applied both within and across users. This approach enables experts to investigate inter-subject variability, assess population-level behaviour models, and explore how behavioural PM can support personalised monitoring and group-level health insights.

Expanding our research to multi-user datasets and developing quantitative measures to assess the effectiveness of this integration are significant directions for future work. These steps will not only strengthen the evaluation of the framework but also enhance its applicability across a broader range of healthcare contexts.

## 6. Conclusions

The paper underscores the potential of innovative behavioural analysis in understanding individual behaviours and tailoring healthcare interventions. By leveraging information from multimodal IoT systems and aggregating the data, we can obtain a comprehensive view of physical health. We have investigated this potential in our study by considering a use case and data collected by a multimodal IoT system, including ambient sensors in the house, a wristband, and a smartphone. The proposed approach for behaviour analysis involves grouping daily routines based on their alignment with health guidelines. These routines are then modelled by process mining techniques and clustered to highlight similarities between daily routines from several days. We also provided calendars and timelines to visualise all extracted information in an understandable format and illustrate how it can be used to discover the relation between several activities and health guidelines. The impact of external events on the behaviour can also be identified. The outcome of behavioural analysis and the resulting comprehensive view can empower health experts to provide more accurate interventions based on actual behavioural patterns. This enhances the potential for timely and effective health monitoring and interventions.

## Figures and Tables

**Table 1:** The location clusters of the Insufficient group.

Cluster	No. of Days	Main Characteristics
Insufficient-L1	38	Bedroom ≈12.5 h, LivingRoom ≈5.5 h, Kitchen ≈3 h and Out-of-Home ≈2 h
Insufficient-L2	6	Bedroom ≈11 h, LivingRoom ≈7.5 h, Kitchen ≈3.5 h and Out-of-Home ≈1 h
Outlier	6	No specific pattern

**Table 2:** The statistics and main characteristics of clusters within the Insufficient group based on activity.

Cluster	No. of Days	Main Characteristics
Insufficient-A1	31	Sleeping ≈8 h, Praying ≈3 h, Watching TV ≈4 h, Cooking ≈2 h and Using phone ≈1.5 h
Insufficient-A2	6	Sleeping ≈8 h, Praying ≈3.5 h, Watching TV ≈5 h, Cooking ≈2 h and Using phone ≈1.5 h
Outlier	13	No specific pattern

**Table 3:** The main characteristics of clusters within the Sufficient group based on location.

Cluster	No. of Days	Main Characteristics
Sufficient-L1	53	Bedroom ≈11.5 h, LivingRoom ≈5.5 h, Kitchen ≈3 h and Out-of-Home ≈3 h
Sufficient-L2	6	Bedroom ≈10 h, LivingRoom ≈4.5 h, Kitchen ≈2 h and Out-of-Home ≈6.5 h
Sufficient-L3	4	Bedroom ≈15 h, LivingRoom ≈4.5 h, Kitchen ≈1.5 h and Out-of-Home ≈2.5 h
Outlier	11	No specific pattern

**Table 4:** The main characteristics of behaviour routines based on activities for the clusters in the Sufficient group.

Cluster	No. of Days	Main Characteristics
Sufficient-A1	55	Sleeping ≈8 h, Praying ≈2 h, Watching TV ≈3.5 h, Cooking ≈1.5 h and Using phone ≈1.5 h
Sufficient-A2	8	Sleeping ≈8 h, Praying ≈1.5 h, Watching TV ≈2.5 h, Cooking ≈2 h and Using phone ≈1.5 h
Sufficient-A3	5	Sleeping ≈8 h, Praying ≈3 h, Watching TV ≈3.5 h, Cooking ≈1 h and Using phone ≈2 h
Outlier	6	No specific pattern

**Table 5:** The main characteristics of location-based clusters in the Desirable group.

Cluster	No. of Days	Main Characteristics
Desirable-L1	15	Bedroom ≈10 h, LivingRoom ≈5.5 h, Kitchen ≈3 h and Out-of-Home ≈4.5 h
Desirable-L2	2	Bedroom ≥8.5 h, LivingRoom ≈2 h, Kitchen ≈3 h and Out-of-Home ≈10 h
Desirable-L3	2	Bedroom ≈10 h, LivingRoom ≈4 h, Kitchen ≈2 h and Out-of-Home ≈7.5 h
Outlier	3	No specific pattern

**Table 6:** The main characteristics of activity-based clusters in the Desirable group.

Cluster	No. of Days	Main Characteristics
Desirable-A1	14	Sleeping ≈7.5 h, Praying ≈1.5 h, Watching TV ≈3 h, Cooking ≈1.5 h and Using phone ≈1.5 h
Desirable-A2	5	Sleeping ≈7.5 h, Praying ≈1 h, Watching TV ≈4 h, Cooking ≈2.5 h and Using phone ≈1.5 h
Outlier	3	No specific pattern

**Table 7:** The number of days based on the groups.

Type of Days	Insufficiant	Sufficiant	Desirable	Total
Regular	2	22	19	43
COVID and lockdown	48	52	3	103
Ramadan	8	21	-	29
New Year Holidays	13	-	-	13

## Data Availability

Data is not available publicly and it is only available for research upon request.
